# Supplementary material for: Monitoring the Establishment of VOC Gamma in Minas Gerais, Brazil: A Retrospective Epidemiological and Genomic Surveillance Study
Source: Viruses. 2022 Dec 9;14(12):2747. doi: 10.3390/v14122747 (PMC9781153; doi:10.3390/v14122747)
Supplement: Supplementary file 1 [file viruses-14-02747-s001.zip › viruses-2025328-supplementary/Supplementary Figure S1.pdf]

Regional Health Units - Minas Gerais

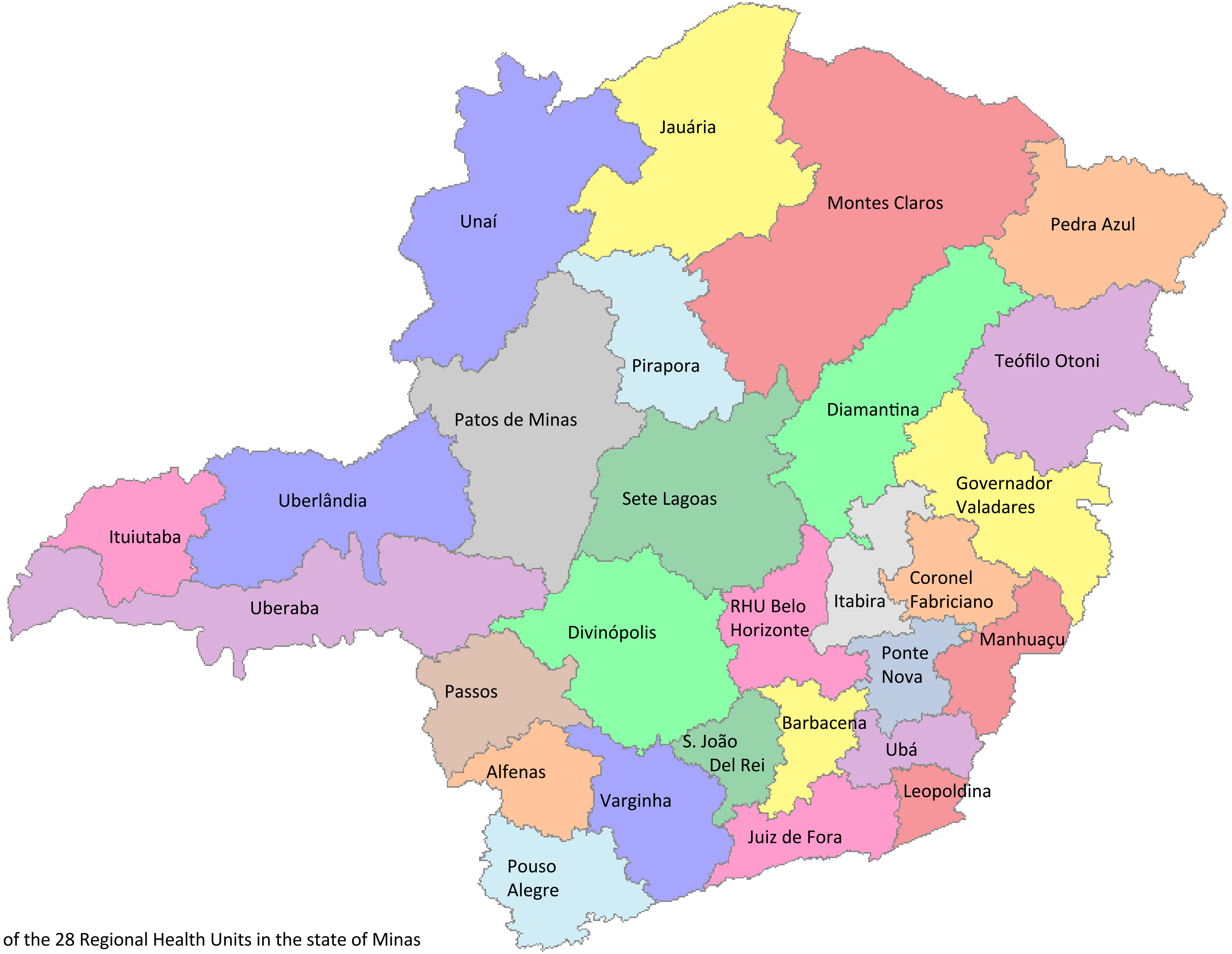

Supplementary Figure S1. Representation of the 28 Regional Health Units in the state of Minas Gerais, distributed among the macro-regions of the state.

Source: Secretaria de Saúde - Minas Gerais
